# The complete mitochondrial genome of the *Cyclemys fusca* (Chelonia: Geoemydidae)

**DOI:** 10.1080/23802359.2018.1481786

**Published:** 2018-07-27

**Authors:** Meng Wang, Yuanhua Lu, Liuwang Nie

**Affiliations:** Life Science College, Anhui Normal University, Provincial Key Lab of the Conservation and Exploitation Research of Biological Resources in Anhui, Wuhu, China

**Keywords:** Complete mitochondrial genome, phylogeny, Cyclemys fusca

## Abstract

The complete mitochondrial genome (mitogenome) of *Cyclemys fusca* was obtained and characterized in this study. The circular molecule is 16,491 bp in length and contained 13 protein-coding genes (PCGs), two ribosomal RNA (rRNA) genes, 22 transfer RNA (tRNA) genes, and one non-coding region (control region). Its gene arrangement type is identical to the type of most vertebrate. All protein-coding genes initiate with ATG as start codon, except for COI started with GTG. Interestingly, COI and ND6 end up with AGG. The complete mitogenome of *C. fusca* provides the basic data to research molecular systematics of Geoemydidea.

The Myanmar brown leaf turtle (*Cyclemys fusca*) is found mainly in India and Myanmar (Rhodin et al. 2017). *Cyclemys fusca* belongs to the genus *Cyclemys* within the family Geoemydidea (Li et al. 2017). At present, only partial mitochondrial genome of *C. fusca* has been published on NCBI (Kundu et al. 2016), the complete mitochondrial genome sequence has not been reported.

The specimen of *C. fusca* (code No. 26080169) was obtained from Shanghai Zoo and stored in Anhui Provincial Key Lab of the Conservation and Exploitation Research of Biological Resources from Anhui Normal University. Total genomic DNA was extracted from the tip tail tissues using the Sangon Animal genome DNA Extraction Kit (Shanghai, China). Mitochondrial genome was amplified with 16 primers using PCR and then sequenced. These primers were designed by Oligo 7.0 based on the complete mitochondrial genome of *C. atripons* (GenBank: EF067858). BioEdit 7.2.3 was used to assist artificial sequence splicing after sequencing (Hall 1999). The mitochondrial genome sequence of *C. fusca* was submitted to GenBank for accession number JX218031.

The whole mitochondrial genome is a circular molecule of 16,491 bp in size and consists of 13 protein-coding genes, two rRNA genes, 22 tRNA genes, and one control region. The overall base composition of mitogenome was A (34.3%), T (27.0%), C (25.7%), and G (13.0%). All the protein coding genes encoded on the heavy strand, except ND6 encoded on the light strand. The lengths of 12S rRNA and 16S rRNA are 965 bp and 1599 bp, respectively. The length of D-loop is 986 bp, ranging from 15,506 to 16,491 bp. All tRNAs harbor the cloverleaf secondary structures predicted by tRNAscan-SE (Schattner et al. 2005) except for the tRNA^-Ser^(AGN) lacking the DHU-arm. All protein-coding genes initiate with ATG as start codon, except for COI started with GTG. Six PCGs (COII, COIII, ND4, ND5, ATP6, and ATP8) stop with usual codon TAA, three PCGs (ND1, ND2, ND4L) stop with codon TAG, two PCGs (ND3, Cyt *b*) stop with incomplete codon TA, while COI and ND6 end up with AGG.

Using two species of the Testudinidae as the outgroups, we performed the maximum-likelihood (ML) and Bayesian inference (BI) phylogenetic analyses to clarify the phylogeny of 21 Geoemydidea species, including the *C. fusca*, based on nucleotide sequence data of 13 PCGs with RAxML version 8 (Stamatakis 2014) and MrBayes 3.2 (Huelsenbeck and Ronquist 2001). As shown in the phylogenetic tree (Figure 1), *C. fusca* and five species of genus *Cyclemys*, formed a monophyletic group, and the genus *Cyclemys* is closely related to genus *Notochelys*, *Heosemys*, and *Sacalia*. This conclusion is consistent with previously reported results (Wang et al. 2012, 2017; Zhou et al. 2015).

**Figure 1.**
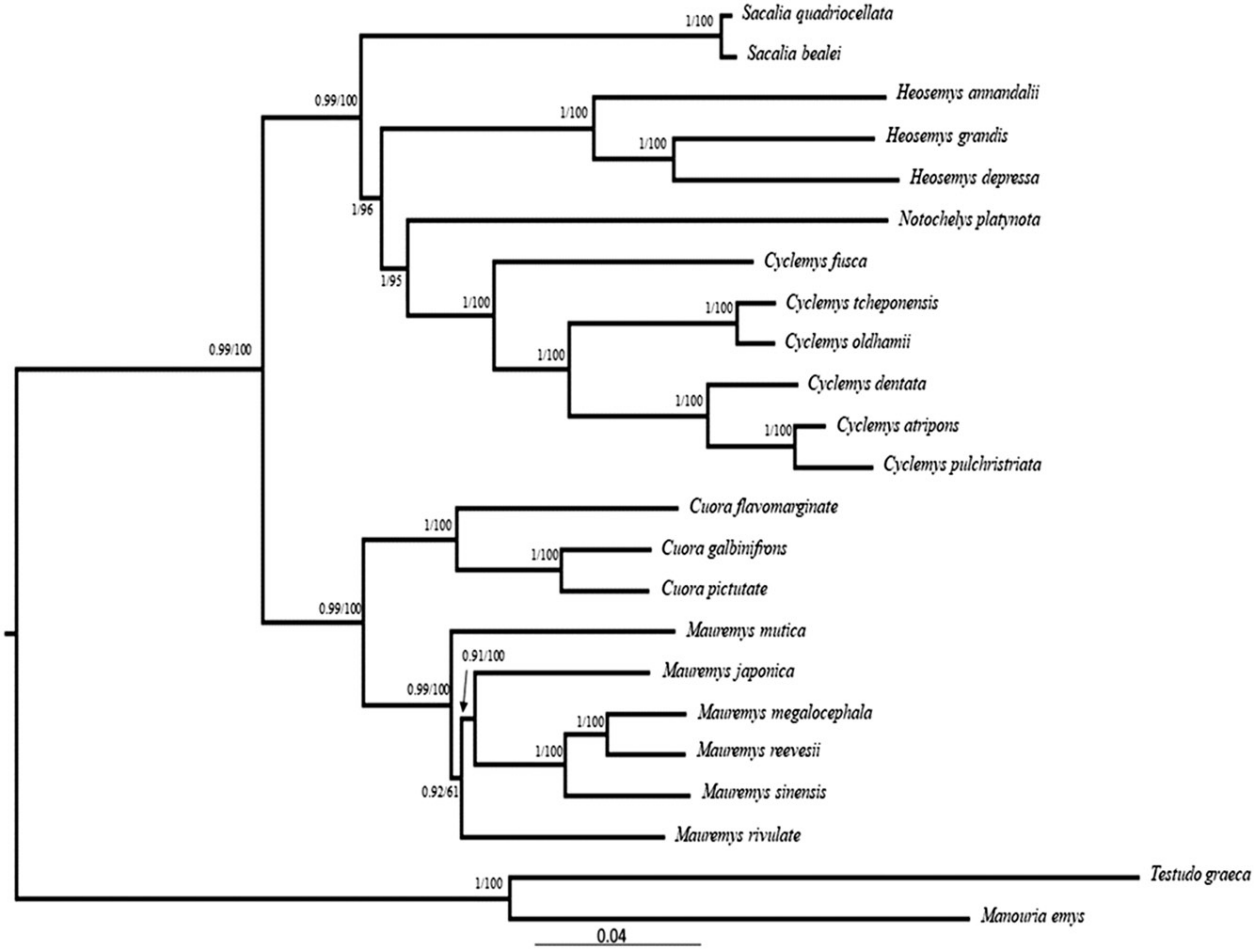
Phylogenetic tree of C. fusca based on the nucleotide dataset of the 13 PCGs. Number above each node indicates the BI/ML bootstrap support values.
